# Supplementary material for: Are carbon emissions trading and green financial instruments synergistic? -Comprehensive quantitative research based on content analysis
Source: PLoS One. 2024 Mar 7;19(3):e0298601. doi: 10.1371/journal.pone.0298601 (PMC10919601; doi:10.1371/journal.pone.0298601)
Supplement: S1 Data — (ZIP) [file pone.0298601.s001.zip › Data/地级市Z_F工作报告.zip!/╣π╬≈/╣≡┴╓╩╨/└┤╫╘╣1⁄2╓┌║┼í╛┬φ┐╦╩2╛▌═°í┐_2019.docx]

各位代表：

　　现在，我代表市人民政府，向大会报告政府工作，请予审议，并请各位政协委员和列席会议的同志提出意见。

　　一、2018年工作回顾

　　刚刚过去的2018年，是全面贯彻落实党的十九大精神的开局之年，是改革开放40周年、自治区成立60周年。我们坚持以习近平新时代中国特色社会主义思想为指导，在自治区党委、政府和市委的正确领导下，坚持稳中求进工作总基调，坚持新发展理念，坚持高质量发展，坚持桂林国际旅游胜地建设“一本蓝图绘到底”，统筹推进稳增长、促改革、调结构、惠民生、防风险各项工作，经济运行总体平稳、稳中提质、稳中向好，各项事业全面进步，主要经济指标增速比2017年有大幅提升，综合实力显著增强，较好完成市五届人大三次会议确定的目标任务。初步预测，地区生产总值增长7.0%以上，财政收入增长7.3%，规模以上工业增加值增长5.2%，固定资产投资增长14.3%，社会消费品零售总额增长9.5%，城镇居民人均可支配收入增长6.7%，农村居民人均可支配收入增长9.8%，居民消费价格上涨2.3%，常住人口城镇化率达50%，节能减排完成自治区下达目标。

　　——政策优势持续叠加。湘江战役长征文化资源保护利用工作得到中央领导的高度重视；继建设国际旅游胜地和国家健康旅游示范基地后，又获批建设国家可持续发展议程创新示范区；自治区即将出台《以世界一流为发展目标　打造桂林国际旅游胜地的实施意见》《桂林漓江生态保护和修复提升工程方案》等为桂林量身定制的重要文件，桂林在全国、全区发展大局中的地位进一步凸显。

　　——新动能加快成长。成功引进会仙湿地国际旅游度假小镇、兴安乐满地5A级文化旅游康养综合体、美好家园国际旅居康养小镇、愚自乐园地中海度假村(二期)、益田(雁山)民国风情小镇、航天科技小镇等一批投资50亿元以上的重大项目；开工建设深科技智能制造、中国中药(桂林)产业园等工业项目，其中深科技项目全部达产后，可年产智能手机5000万部；福达、平钢、桂康、新桂轮等重点企业技改步伐加快，达产后将实现产值200亿元以上。

　　——交通优势更加凸显。两江国际机场T2航站楼建成使用，机场年旅客吞吐能力增至1200万人次；桂林航空公司持续壮大，机队规模扩充至11架，累计开通航线70余条，形成辐射全国主要旅游城市的航空网；在全区率先实现与香港高铁“一乘直达”，桂林区域性交通枢纽地位更加突出。

　　——营商环境持续优化。出台系列政策措施；累计为企业减负超20亿元；市本级99.4%的依申请权力事项和公共服务事项实现“最多跑一次”，组建不动产登记和房产交易中心，不动产登记由平均18个工作日压缩至7.5个工作日，得到自治区党委、政府主要领导的批示肯定，全区推进“一事通办”[1]改革工作现场会在我市召开。

　　——旅游产业快速发展。建成运行“一部手机游桂林”平台，开启智慧旅游新时代；“桂林千古情”开业以来持续火爆，月均接待游客35万人次，成为文化旅游新亮点；桂林连续入选春节、端午、国庆10大国内热门旅游目的地，高票登榜央视“2018最美揽夏地”，全年接待游客突破1亿人次，旅游总消费超1300亿元，分别增长32.6%、43.2%。

　　——脱贫攻坚卓有成效。筹措财政扶贫资金18.59亿元，预计2018年龙胜、资源2个贫困县摘帽，164个贫困村出列，6.48万贫困人口脱贫，贫困发生率降至2.2%以下，形成“生态循环农业+扶贫”“旅游+扶贫”“电商+扶贫”等产业扶贫新模式，实现100%的贫困村、80%的贫困户都有特色支柱产业，发展后劲明显增强。

　　——平安建设成绩斐然。社会治安明显改善，刑事警情下降40%，创十年来新低；扫黑除恶专项斗争取得重大成果，打击传销初见成效，大型活动安保有力；平乐社区戒毒模式全国推广，群众安全感持续提升，为国际旅游胜地建设提供了有力保障。

　　——桂林名片更加亮丽。圆满完成庆祝自治区成立60周年桂林系列活动，新型城镇化示范乡镇建设、以项目带动旧城改造、生态文化旅游融合发展等“桂林经验”“桂林模式”得到中央代表团的高度肯定；桂林入选中宣部“庆祝改革开放40年·百城百县百企调研行”名录，被中央媒体集中报道；全国运输服务与旅游融合发展现场会、全国乡村绿化美化现场会在我市举行，“桂林山水甲天下”的金字招牌更加闪亮！

　　一年来，我们主要做了以下工作：

　　(一)突出“1+2”国家战略[2]，发展优势持续增强

　　国际旅游胜地建设加快推进。全面落实自治区“以世界一流为发展目标，打造桂林国际旅游胜地”工作要求，旅游产业用地等政策有效实施，离境退税政策落地，胜地建设升级发展相关政策争取工作取得积极进展，两江四湖、龙脊梯田、资源八角寨改造提升等规划纲要确定的重大项目顺利推进，雁山草坪等26个特色旅游小镇加快建设，“一轴两带”[3]的产业发展格局加速形成，产业结构进一步优化，第三产业增加值占地区生产总值比重达49.2%，胜地建设中期评估顺利完成。

　　国家可持续发展议程创新示范区建设开局良好。自治区、市、县(区)三级均成立了创新示范区建设工作领导小组，形成上下联动、协同推进的工作格局。《桂林市可持续发展规划(2017—2030年)》《桂林市国家可持续发展议程创新示范区建设方案(2017—2020年)》顺利实施。成功召开国家可持续发展议程创新示范区推进大会，举办2018中国—东盟可持续发展创新合作国际论坛，争取到国家、自治区科技项目800多项，创新示范区建设进展顺利。

　　国家健康旅游示范基地建设成效明显。崇华中医街获评全国首批中医药健康旅游示范基地，夕阳红养老中心等示范项目成效显现，魅力花园国际养老公寓正式运营，翠竹孝慈轩养老服务中心基本建成，桂林工人疗养院永福基地等项目顺利推进，恭城平安康养特色小镇等项目开工建设，“漓水青山·养生桂林”城市品牌进一步打响。

　　(二)突出高质量发展，工业发展稳步向好

　　抓园区建设。园区土地收储面积大幅提升，新建标准厂房75万平方米，超过过去三年建成面积总和，产业承载能力显著增强。高新区实施腾讯众创空间等重大项目14个，总投资124亿元；经开区成为全区首个获得国家认证的绿色园区，引进广西建工装配式建筑等重点项目21个，总投资225.3亿元；高铁经济产业园挂牌成立，完成基础设施投资1.76亿元，修建道路7条，智慧产业园孵化中心等项目加快推进；荔浦光电产业园、平乐中联智能科技产业园、灌阳石材产业园、全州米粉产业园等特色产业园区建设进展顺利，全市产业园区优势互补、竞相发展格局基本形成。

　　抓重点产业。初步统计，电子信息、先进装备制造、医药及生物制品、生态食品四大主导产业总产值占全市工业总产值比重达45.9%；围绕重点产业，实施亿元以上工业项目50个，其中5亿元以上项目16个，比亚迪产业基地主体工程基本完工，晨天恒源高性能合金项目建成投产。

　　抓企业服务。坚持领导干部联系服务企业制度，出台推动工业高质量发展系列政策，着力解决企业融资、用工、用地、审批等方面的困难，深科技智能制造项目两个月内实现签约开工，刷新“桂林速度”；福达、燕京漓泉、国际线缆等重点骨干企业快速发展，电力电容器等4家企业入选2018广西最具竞争力民营企业十强，数量居全区第一。新培育规上工业企业59家。

　　抓创新支撑。高技术产业总产值增长18.9%，高新技术企业保有量突破200家；三金药业荣膺第三届中国质量奖提名奖，桂林南药荣获第四届自治区主席质量奖；智神公司稳定器产品摘得全球工业设计顶级奖项，云璟科技、蓝港科技获全国创新创业大赛优秀企业奖；君泰福电气、广陆数控等6家企业获评广西智能工厂示范企业，数量居全区首位。

　　(三)突出乡村振兴，“三农”基础更加坚实

　　加快发展现代特色农业。农林牧渔业增加值增长5.0%。突出综合生产能力提升，粮食总产量185.85万吨，水果产量全区领先；桂柳家禽成为国家农业产业化重点龙头企业，新增农民专业合作社870家、家庭农场393家；农业耕种收综合机械化水平达60.8%。突出规模效应，促进现代特色农业示范区增点扩面提质升级，自治区级核心示范区增至29个，各级示范区(园、点)总数达959个。突出品牌强农，新认证“三品一标”[4]产品43个，荔浦芋、恭城月柿、永福罗汉果纳入广西首批区域公用品牌目录。突出农旅融合，新增自治区休闲农业示范点6家，乡村旅游接待游客3200万人次。

　　加快完善农村基础设施。农田水利设施、水土保持综合治理等项目稳步推进，新增高效节水灌溉面积5.16万亩，建成农村饮水安全巩固提升工程272处。建制村通畅率达99.8%，乡镇和建制村通公交率分别达71.6%和40.8%，全区“四好农村路”[5]建设现场会在荔浦召开。村级公共服务中心覆盖率达80.8%，乡村教育、卫生、文化、体育等公共服务能力显著提升。

　　加快推进“美丽桂林”乡村建设。完成农村改厨改厕14.7万户、改圈7895户。31个传统村落保护发展示范村建设基本完成，118个乡土特色示范村(带)、88个“美丽桂林·宜居乡村”示范村加快建设，获自治区级“美丽乡村”示范村18个、绿色村屯81个。

　　(四)突出旅游带动，三产发展提质增效

　　实施“旅游+”战略，带动现代服务业转型升级，第三产业增加值增长8.9%，对全市经济增长贡献率达58.2%，占税收比重超60%，成为拉动经济增长的主动力。

　　推进旅游业提档升级。狠抓融合发展，大力推进旅游与生态文化、康养医疗等产业融合，融创万达文化旅游城等一批融合发展项目加快建设，以桂林文化旅游大道为主轴的旅游新“航空母舰”加快打造，旅游新业态不断涌现。狠抓旅游“双创”[6]工作，秀峰成为首批自治区级全域旅游示范区，全州、永福、灌阳、平乐成为自治区全域旅游示范区创建单位，荔浦、资源、雁山跻身广西特色旅游名县“双创”工作持续走在全区前列。狠抓旅游品牌建设，平乐温德姆酒店建成运营，香樟华苹度假酒店基本建成，香格里拉、悦榕庄等酒店业务快速增长。狠抓景区景点建设，新增国家4A级景区6家、自治区级生态旅游示范区4家，漓江东岸百里生态示范带和西岸百里休闲示范带新景观加快打造，兴坪休闲养生度假区等重大项目顺利推进，全州天湖度假区滑雪场建成营业。狠抓旅游基础设施建设，桃花湾旅游休闲绿道投入使用，龙脊梯田大循环公路等项目加快推进，新建旅游厕所113座。狠抓旅游管理提档升级，兴安成为第四批全国旅游标准化试点县创建单位；开展旅游消费购物市场整治、旅行社清理整治等专项行动，注销“僵尸旅行社”21家；建设旅游购物企业监管平台，开展旅游诚信建设，市场秩序持续向好。狠抓旅游营销推介，与国内外知名媒体开展旅游宣传合作，加入“北上陕”中国入境旅游枢纽合作机制[7]，恢复开通桂林至曼谷直飞包机，新开通“中国桂林—越南下龙”跨国自驾游路线。

　　推进服务业量质齐升。新增规上限上服务业企业128家，23个服务业集聚区投入运营。6个国家电子商务进农村综合示范县建设稳步推进。成功举办2018华为物流峰会暨物流技术论坛，京东智能物流“无人机”广西首飞仪式在全州举行，中辰电商物流交易中心、苏桥无水港项目建设进展顺利，物流配套体系更加完善。全年机场旅客吞吐量近900万人次，铁路旅客发送量1682万人次，同比增长8.8%，到达量1673万人次，同比增长7.9%，运输收入同比增长19.1%；邮政业务总量增长37%，电信业务总量增长220%。金融机构贷款余额增长17.4%。开展桂林米粉北京产销对接活动，大力发展保鲜速食型米粉，米粉销售收入近70亿元。初步预计，批发、零售业商品销售额分别增长12.1%、9.7%，住宿、餐饮业营业额分别增长10.4%、19.3%。桂林航旅、桂林国旅、桂林百货等企业快速发展，微笑堂、漓江大瀑布、大公馆、中石油桂林公司、中石化桂林公司等企业成为服务业稳增长的重要力量。

　　(五)突出项目建设，城乡面貌深刻变化

　　坚持项目带动战略，落实“一联三”[8]“五个一”[9]等工作机制，全年实施重大项目990项，完成投资986亿元，有力推动城乡协调发展。

　　重大基础设施建设快速推进。高铁五通站正式启用；阳朔至鹿寨高速公路复工，贺州至巴马(桂林段)、荔浦至玉林、灌阳经恭城至平乐、桂林至柳城高速公路加快推进，新增10条高速公路纳入全区高速公路网规划；国道322线兴安绕城公路等项目完工，新增等级公路通车里程424公里。桂林市防洪及漓江补水枢纽工程三座水库全部下闸蓄水，长塘水库设计方案通过国家审查。兴安石板岭等6个风电项目竣工验收，县县通天然气等工程有序推进。

　　中心城市功能品位持续提升。坚持“新区产城互动做加法，老城疏解提升做减法”，市区面积135平方公里，常住人口128万人，城市绿化覆盖率达40.04%。老城疏解提升成效明显，汽车总站完成搬迁，中隐路侯山隧道双向通车，万福路、相人山路改造等工程顺利完工，16座人行天桥投入使用；漓江城市段两岸片区改造提升等项目加快建设，11个特色街区改造项目顺利完成，第二批历史文化标识全面建成，“一城文化满城绿”桂林风韵不断彰显。临桂新区功能加快完善，建成面积达30平方公里，常住人口35万人。实施建设项目123项，完成投资86亿元，建设大厦、发展大厦、金融大厦建成使用；桂林师专实现整体搬迁，桂林中学(临桂校区)、博元小学等学校正式启用；旅游综合医院、吾悦广场、枫林市场等项目加快推进；新区环城水系全线贯通，新龙路桥等5座桥梁建成通车，“七纵九横”主干路网和“山环水绕”山水新城格局基本成型。城市精细化管理水平显著提升，实施《桂林市城市市容和环境卫生管理条例》，坚持最严城市管理绩效考评，继续开展创城“金点子”活动，统筹推进创卫复审、文明城市创建等工作，拆除违法建设276万平方米、违法户外广告牌3.5万平方米，打击非法燃气经营点490家；新增垃圾分类试点小区23个，山口生活垃圾焚烧发电项目建成运行；数字化城市管理系统覆盖六城区及阳朔县城、灵川八里街片区；通过国家园林城市复查，文化品位休闲之都魅力绽放。

　　新型城镇化示范乡镇建设全面提速。第四批16个新型城镇化示范乡镇、7个自治区百镇建设示范工程顺利推进，2个国家级、6个自治区级新型城镇化示范县加快建设，阳朔福利镇获评中国历史文化名镇，灵川大圩等5个镇入选广西第一批特色小镇培育名单，数量居全区首位。

　　县域经济发展活力进一步增强。荔浦撤县设市，阳朔、荔浦、龙胜被评为广西科学发展先进县，临桂连续三年入选全国投资潜力百强区，全州、阳朔、灵川等县城新区加快建设，恭城、兴安康养旅游融合发展，龙胜、资源、灌阳脱贫攻坚成效明显，临桂、永福服务新区、经开区建设成效显著，平乐园区建设势头强劲，桂林“城乡一体、生态美丽、文化繁荣、富裕和谐”大家园展现出独特魅力。

　　(六)突出生态文化，桂林形象日益彰显

　　强化漓江科学保护。持续推进漓江“三统”改革[10]，完成漓江游览30年来规模最大的游船提档改造，平乐至阳朔等一批分时分段游航线陆续开通，漓江旅游品质大幅提升。强化水源林保护，漓江上游水库群实施联合调度，科学补水，漓江涵养功能持续提升。强力推进截污治乱，完成漓江截污(二期)收尾工程，“四乱一脏”[11]专项整治持续推进。加强基础设施建设，两江四湖(二期)连通水系通航，建成漓江城市段东岸慢行步道(一期)及西岸历史文化长廊，阳朔杨堤等一批旅游码头投入使用，基本完成伏龙洲生态修复，漓江两岸新景观休闲旅游带加速形成。漓江活动壅水科学试验坝开展常态化壅水，常年呈现出青山环绕、碧水长流的美丽景观。

　　加强生态环境治理。严格落实党政同责、一岗双责，打好污染防治攻坚战，扎实推进中央环保督察反馈意见整改。制定实施大气、水、土壤污染防治攻坚三年计划《桂林市销售燃放烟花爆竹管理条例》经市人大常委会审议通过，在全区率先开展机动车排放检验机构标准化建设，市区空气质量优良天数达324天，同比增加16天，细颗粒物(PM2.5)平均浓度值同比下降13.6%，恭城获全国首个气候宜居县；全面落实河(湖)长制，南溪河、灵剑溪和道光水等城市黑臭水体整治全面完成，城市污水集中处理率近100%，主要河流考核断面水质达标率100%；完成农用地土壤污染详查。采石场规范化建设进展顺利，第一批7家试点采石场完成出让。国家生态文明先行区建设有序推进，水生态文明城市建设试点和绿色交通城市创建高分通过国家验收，荔江、龙脊梯田、天湖、灌江国家湿地公园加快建设，新增自治区级生态县(市、区)5个、生态乡镇5个，完成植树造林22.92万亩，全市森林覆盖率提高到71.23%。

　　抓好文化传承创新。全面做好湘江战役红军遗骸收殓保护，推进长征文化资源保护利用。持续唱响历史文化保护利用“三部曲”，桂林文化旅游中心启动建设，逍遥楼、正阳东西巷、靖江王府片区历史文化旅游休闲街区成为传承历史文脉新地标。靖江王府维修工程顺利完工，靖江王陵散葬民坟治理工程(一期)基本完成，迁移民坟685座。灵渠成为广西首个“世界灌溉工程遗产”，龙脊梯田系统入选全球重要农业文化遗产。文化精品创作成果丰硕，民族歌剧《刘三姐》在国家大剧院成功首演，桂剧《破阵曲》获广西戏剧展演桂花金奖，《桂林历史文化大典》出版，《桂林有戏》受到中外游客广泛欢迎。文化惠民成效显著，“百姓大舞台”等群众文化品牌活动常办常新，171家文化场馆实现免费开放。文化产业发展取得新成效，原创公益剧《可可小爱》亮相全国大众创业万众创新活动周，象山文化产业园入选自治区文化产业示范园区，两江四湖旅游公司入选自治区文化产业示范基地。

　　(七)突出改革创新，发展活力更加强劲

　　推进重点领域改革。市级层面89项改革任务、23项重点改革专题基本完成。深化“放管服”[12]改革，全面推行“证照分离”改革，实现“多证合一”“容缺受理”，全程电子化业务量占全区80%以上，新增市场主体5.6万户，增长22.6%。机构改革有序推进。加快推进漓江管理体制改革，出台《桂林漓江风景名胜区水上游览项目经营权管理办法》《桂林漓江风景名胜区条例》(草案)提请市人大常委会审议，桂林景区旅游管理公司组建运营；413所学校参与义务教育学区制管理改革；桂林成为广西唯一医联体建设整体推进改革试点城市，灌阳医改模式获国家激励通报；农村改革稳步推进，土地承包经营权确权登记颁证率达95.3%，新增土地流转面积21万亩。第四次全国经济普查工作稳步开展，新时代基层统计工作规范化建设扎实推进。

　　不断提升科技创新能力。桂林电子科技大学“卫星导航定位与位置服务”实验室成为广西唯一由高校建设的国家地方联合工程研究中心，新增国家认定企业技术中心2家，荔浦成为国家首批创新型县(市、区)。每万人口发明专利拥有量居全区前列，获评中国专利优秀奖3件；获广西科学技术特别贡献奖1项，包揽2项自然科学一等奖。石墨烯研究最新进展国际会议在桂林举办。建立广西地级市首个海内外高端人才创业创新示范基地，新建院士工作站4家，新入站两院院士4人。

　　打好防范重大风险攻坚战。抓好金融领域风险防控和处置化解工作，坚决打击非法集资、恶意逃废债务等违法违规行为。采取压缩预算支出、推进城镇低效用地再开发、加快土地出让、处置闲置资产等方式，化解债务存量，严控债务增量，防范政府债务风险。

　　扩大对外合作。引进内外资项目212个，合计到位资金780.9亿元。成功举办旅游趋势与展望国际论坛、中国—东盟博览会旅游展、桂林国际山水文化旅游节。与韶关等6市签署红色旅游合作“桂林宣言”。

　　(八)突出脱贫攻坚，民生事业加快发展

　　民生领域支出占一般公共预算支出总额的78.9%。自治区层面、市级层面为民办实事项目年度任务全面完成。

　　坚决打赢脱贫攻坚战。深入开展“七个一批”[13]“六大行动”[14]，推进基础设施建设大会战，新建或升级改造贫困村道路1358条，改造农村贫困户危房6272户。开展特色产业扶贫，所有贫困村村级集体经济收入3万元以上。大力推进就业扶贫，2.39万贫困人口实现稳定就业。电商扶贫成效显著，3000多户贫困户通过电商实现增收。实施易地扶贫搬迁集中安置项目18个，完成易地扶贫搬迁8163人。教育扶贫、健康扶贫、肇庆—桂林扶贫协作深入推进，社会扶贫成效显著。

　　加强社会保障。城镇新增就业5.49万人，农村劳动力转移就业12.49万人次。“五险”[15]参保人数738.84万人次，发放城乡低保资金5.26亿元。开工建设棚户区改造住房1.18万套，基本建成保障性住房8332套，农村危房改造新开工1.28万户。

　　推进社会事业协调发展。加快发展教育事业，学前教育三年毛入园率、九年义务教育巩固率、高中阶段教育毛入学率分别达87.2%、98.1%、91.5%；实现自治区级义务教育发展基本均衡县(市、区)全覆盖，累计15个县(市、区)通过国家评估认定；投入资金12.61亿元，新建、改扩建学校347所，新增学位2.69万个，恢复十八中、中山中学初中部办学。市卫校雁山校区、桂林市示范性综合实践基地学校投入使用，桂林生命与健康职业技术学院正式招生；高校集聚区建设加快推进，花江智慧谷电子信息创业产业园、芳香东路建设进展顺利；雁山大学集中区中心环线全线贯通，雁山人民医院竣工验收。全面推进健康桂林建设，市中医药传承创新工程开工建设。全民健身活动广泛开展，成功举办环广西公路自行车赛、桂林国际马拉松赛、资源国际漂流精英挑战赛、广西第九届残运会暨第四届特奥会等重大赛事。

　　加强和创新社会治理。安全生产、食品药品安全监管得到加强。信访维稳实现根本好转，全区信访工作现场经验交流会在桂林召开，社会大局和谐稳定。

　　一年来，我们全面加强自身建设，政府治理效能进一步提升。坚持依法行政，积极推进法治政府建设，健全重大行政决策机制，实施重大决策预公开，加强重点领域的审计监督，依法接受市人大及其常委会的监督，自觉接受市政协的民主监督，人大代表建议和政协提案办结率达100%。认真学习贯彻中央、自治区和市委全面从严治党各项决策部署，切实履行政府系统党风廉政建设主体责任，狠抓中央八项规定精神和国务院“约法三章”落实，持之以恒纠“四风”，组织开展形式主义、官僚主义突出问题专项整治活动，健全厉行节约长效机制，“三公”经费下降3.1%，严厉查处重点领域腐败和违规违纪问题，反腐败斗争取得压倒性胜利。

　　一年来，国防教育深入开展，国防动员、人民防空、民兵预备役和双拥、优抚安置等工作进一步加强，军民融合深入发展。民族宗教、社会科学、地方志、决策咨询、外侨、对台工作、编制、人事、质监、物价、国资国企、机关事务、接待、档案、供销、保密、口岸、气象、水文、测绘、海关、消防、防震减灾、老龄事业、公共机构节能等工作取得新进步，中直、区直驻桂林单位取得新成绩，工会、共青团、妇联等群团组织在经济社会发展中发挥了重要作用。

　　各位代表！这些成绩的取得，是市委总揽全局、科学决策的结果，是市人大及其常委会和市政协监督支持的结果，是全市各族干部群众团结拼搏、开拓进取的结果。在此，我代表市人民政府，向全市各族人民，向人大代表、政协委员，向各民主党派、工商联、无党派人士、各人民团体和各界人士，向离退休的老领导、老同志，向中央和自治区驻桂林单位，向驻桂林部队和武警官兵，向所有关心支持桂林发展的海内外朋友，表示衷心的感谢！

　　我们也清醒地认识到，我市经济社会发展还面临不少困难和问题：发展不平衡、不充分问题依然突出；稳增长压力依然较大；工业高质量发展任务艰巨；财政收支矛盾较为突出；民生领域尚有短板，等等。我们一定正视这些问题，采取有力措施，切实加以解决。

　　二、2019年工作总体要求和目标任务

　　今年是新中国成立70周年，是实现我市“两个建成”目标的关键之年，做好今年的工作事关长远，至关重要。

　　党的十八大以来，我市经济社会持续健康发展，交通基础设施大幅改善，城乡面貌发生深刻变化，人民群众的幸福感、获得感、安全感显著提升，受到各级领导和广大人民群众的充分肯定和高度评价，为桂林加快发展奠定了坚实基础。习近平总书记“建设壮美广西　共圆复兴梦想”的题词，对湘江战役长征文化资源保护利用工作的高度重视，给我们巨大鼓舞。自治区党委、政府提出以世界一流为发展目标，打造桂林国际旅游胜地，全力推动漓江生态保护和修复提升工程纳入国家项目库，即将出台系列文件，给我们强大支持。新一轮东部产业转移，数字桂林加快建设，为我市实现高质量发展带来重大机遇。

　　今年政府工作的总体要求是：以习近平新时代中国特色社会主义思想为指导，深入贯彻党的十九大和十九届二中、三中全会精神，以及中央庆祝改革开放40周年大会、中央经济工作会议精神，全面落实自治区党委十一届五次全会、全区经济工作会议和市委五届五次全会决策部署，按照建设壮美广西、共圆复兴梦想的总目标总要求，持续深入落实“三大定位”新使命和“五个扎实”新要求，统筹推进“五位一体”总体布局，协调推进“四个全面”战略布局，坚持稳中求进工作总基调，坚持新发展理念，坚持推动高质量发展，坚持以供给侧结构性改革为主线，坚持桂林国际旅游胜地建设“一本蓝图绘到底”，全面深化改革、扩大开放，着力推动国家战略实施，着力推进工业振兴和乡村振兴，着力打好三大攻坚战，着力推进基础设施建设、生态文明建设、文化建设、城乡建设，扩大有效投资，培育发展新动能，努力营造一流营商环境，切实保障和改善民生，保持经济持续健康发展和社会大局稳定，为实现桂林“两个建成”打下决定性基础，为建设壮美广西、共圆复兴梦想作出更大贡献，以优异成绩庆祝新中国成立70周年。

　　今年经济社会发展主要预期目标是：地区生产总值增长7.0%—7.5%，财政收入增长6.5%，规模以上工业增加值增长7%，固定资产投资增长10%以上，社会消费品零售总额增长10%，城镇居民人均可支配收入增长7.5%，农村居民人均可支配收入增长10%，城镇登记失业率控制在4.5%以内，居民消费价格涨幅控制在3.5%左右，常住人口城镇化率提高1.1个百分点，主要节能减排指标控制在自治区下达目标范围以内。

　　实现上述目标，要抓好以下工作：

　　(一)坚持“1+2”国家战略引领，构筑发展新优势

　　强力推进国际旅游胜地升级发展。争取自治区尽快出台《以世界一流为发展目标　打造桂林国际旅游胜地的实施意见》等系列文件，争取设立综合保税区、扩大入境免签旅游团范围、放宽出境口岸限制等政策，加快推进融创万达文化旅游城、兴安乐满地5A级文化旅游康养综合体、兴坪古镇等项目，建设世界一流的旅游目的地，打造美丽和谐的生态环境、高效通畅的综合交通枢纽、绿色高端的现代产业体系、宜居宜游的魅力城乡，构建充满活力的体制机制，力争第三产业增加值占地区生产总值比重超50%，确保2020年基本建成国际旅游胜地，并向更高层次升级发展。

　　强力推进国家可持续发展议程创新示范区建设。深入开展自然景观资源保育等“五大行动”[16]，实施水生态系统保护与修复等17项工程，重点推进喀斯特世界自然遗产地生态景观修复、桂林自然资源生态环境综合地质调查等项目，在景观资源科学保护利用、生态产业融合发展、城乡环境综合治理等方面打造一批城乡可持续发展典范。争取自治区出台推进创新示范区建设的政策。加强可持续发展国际合作交流，办好中国—东盟可持续发展创新合作论坛，力争列入中国—东盟博览会议程并永久落户桂林。

　　强力推进国家健康旅游示范基地建设。统筹推进示范基地建设与胜地升级发展。加快建设桂林国际智慧健康产业园、中国—东盟友好疗养基地等健康旅游产业集聚区，持续推进龙光国际健康旅游休闲养老特色小镇、美好家园国际旅居康养小镇、市中医药传承创新工程、恭城平安康养特色小镇等项目，开工建设会仙湿地国际旅游度假小镇等项目，抓好桂林智慧健康云建设，提升“漓水青山·养生桂林”城市品牌。

　　(二)坚持高质量发展，加快工业振兴

　　工业是我市的短板和弱项，也是加快发展的潜力和关键所在，必须把工业作为全市工作的重中之重。按照“强龙头、补链条、聚集群”的要求，突出抓好龙头企业培育和重大项目带动，实现培育一个“龙头”，带动一个行业，发展一片区域。

　　着力壮大骨干企业。完善“一企一策”帮扶机制，推动各类政策、资源、要素向发展意愿较强、在行业中有一定地位、示范带动作用明显的骨干企业集中。支持企业依托自身优势，向上下游延伸，向系列化发展，扩大生产规模、抢占更多市场；支持企业研发和引进先进技术、先进工艺、先进设备，实现生产标准提升和产品升级换代；支持企业加强质量管控，提升服务质量，开展精准营销，打造一流产品、争创一流品牌；推动福达、国际线缆、三金药业、力源粮油等企业加快成长为区域性龙头企业和行业领军企业。

　　着力培育创新型企业。继续实施高新技术企业倍增、“瞪羚企业”培育计划，推动财政专项资金、产业投资基金、直接股权投资向技术含量高、经济效益好、市场潜力大的创新型企业集中。开辟企业融资“绿色通道”，推动符合条件的企业挂牌上市融资，支持已上市企业增发股票再融资，鼓励更多优质企业发行各类债券开展直接融资；大力支持企业引进战略投资者，与国内外知名企业、高校、科研院所开展深度合作，实现借力发展；大力解决企业人才引进、配套公共服务等方面的问题，推动光隆光电、智神、飞宇等企业跨越发展，迅速成长为行业龙头企业。

　　着力招大引强。牢固树立“以资源换产业，以市场换产业”的理念，依托桂林名城优势、政策优势、驻桂林高校校友资源优势，以及完善的标准厂房配套条件，重点瞄准国内外500强和行业领军企业开展精准招商，做到“无中生有”。要完善主要领导抓招商“招大引强论英雄”的工作机制；要加强招商信息搜集，及时掌握行业重点企业产业转移或投资动向，持续做好跟踪对接；要敢于让利优惠，创造吸引企业入驻的比较优势；要善于依法运用政府资源，解决企业用工、用地、市场等问题，提高招商引资成功率。

　　着力培育规上企业。用好资源整合、产业扶持、财政奖补、政府采购、评先评优等政策手段，培育规上工业企业50家以上。

　　着力抓投入上项目。全面实施“工业项目突破年”行动，全年实施投资1000万元以上重点工业项目110个，总投资260亿元，其中亿元以上项目60个，年度完成投资80亿元，确保今年全市重中之重项目中，工业项目占比达50%以上。重点推进中国中药(桂林)产业园、桂林装配式建筑产业园、荔浦光电产业园、平乐中联智能科技产业园等项目建设，推动深科技智能制造项目(一期)、平钢年产120万吨技改等项目建成投产。通过实施以上重点项目，力争新增工业总产值50亿元，新增税收4亿元以上。

　　着力补链条聚集群。打造优势产业链，依托华为、深科技等企业，打造网络通信设备、智能终端、智能家居等产业链；依托比亚迪、新桂轮等企业，打造新能源汽车及零部件产业链；依托桂林南药、八加一、优利特等企业，打造生物医药、医疗器械等产业链；依托燕京漓泉、莱茵生物等企业，打造酒类、果蔬等特色健康食品产业链。培育新兴产业链，积极培育节能环保装备、健康养生器材、教育信息化装备、教育应用软件等产业，打造节能环保、大健康、现代教育产业链。推进产业协作，围绕本地龙头企业，积极引进上下游配套企业，加快形成全产业链发展格局；鼓励企业参加各类展销活动，推动桂林产品进入国内外大型企业供应网络。

　　着力打造园区主战场。优化园区功能定位，高新区要着力提升创新支撑产业发展能力，打造电子信息和生物医药高端产业生产基地、创新创业先行基地、产学研融合发展基地，力争规模工业总产值超250亿元；经开区要实施三年行动计划，打造成为千亿元园区，形成百亿元华为产业合作区、百亿元罗汉果小镇、百亿元橡胶产业、百亿元新能源汽车产业，力争规模工业总产值超260亿元；高铁经济产业园要大力发展商贸物流业，推动电子信息、高端装备制造等新型工业与现代物流业融合发展，确保规模工业总产值超100亿元。各县(市、区)产业园区要完善产业规划，加快特色园区建设，实现差异化、特色化、集约化发展。完善基础设施，大力推进园区基础设施攻坚行动，继续实施土地收储考核、标准厂房建设奖补政策，进一步加大园区土地收储力度，力争建成标准厂房100万平方米。加快产城融合，重点推进经开区与临桂、永福融合发展，大力完善各类产业园区人才公寓、文化教育、医疗卫生等配套设施，营造良好的工作生活环境。

　　着力抓好工作落实。抓紧制定工业振兴两年行动计划，明确目标任务，压实各县(市、区)、各园区工业发展责任；建立健全工业经济运行“一月一调度”工作机制，及时协调解决重大问题；加快完善工业高质量发展考评体系和奖惩办法，推动资金、资源向工业倾斜。大力弘扬企业家精神、工匠精神，选树和表彰一批企业家先进典型，在全市营造齐心协力振兴工业的浓厚氛围。

　　(三)发展现代农业，推动乡村振兴

　　实施乡村振兴产业发展专项行动。推进“10+3”现代特色农业产业高质量发展三年提升行动[17]，开展粮食绿色高质高效创建工作，大力发展富硒农业、有机循环农业等特色效益农业大产业；新增认证“三品一标”产品10个以上，创建恭城月柿国家特色农产品优势区，培育农产品区域大品牌。加快现代特色农业示范区增点扩面提质升级，力争新创建各级各类示范区(园、点)700个以上，创建一批国家级现代农业园区。推动一二三产融合发展，重点做大月柿、金桔、金槐等特色农产品加工业，打造一批绿色种养、农产品深加工、休闲观光为一体的特色小镇；大力发展冷链物流，推进海吉星农产品物流园等项目建设，构建覆盖城乡、快捷智慧的冷链物流体系；加快发展城郊现代农业，建设一批高端智能大棚、绿色采摘园，培育农耕体验、果蔬采摘、生态餐饮等农旅融合新业态，加快叠彩漓江·都市田园等田园综合体建设；创建5家以上自治区级休闲农业与乡村旅游示范点，打造桂林休闲农业国际旅游示范区。扶持壮大新型农业经营主体，争取新增自治区农业龙头企业10家，培育市级以上农业产业化龙头企业、专业合作社示范社、家庭农场示范场90家。

　　实施基础设施能力提升专项行动。继续实施农村饮水安全巩固提升工程、小型农田水利工程、大中型灌区节水配套改造等农村水利基础设施建设，大力推进高标准农田建设。深入推进“四好农村路”建设，新增村际联网路200公里以上，实现建制村通公交率达43%。推进乡村风貌提升三年行动，建设基本整治型村庄3849个、设施完善型村庄322个、精品示范型村庄32个；做好第四批中国传统村落保护与发展工作。

　　实施农村人居环境整治三年行动。学习借鉴“千村示范、万村整治”工程经验，扎实开展“清洁乡村”巩固“厕所革命”推进、污水治理推进、乡村特色提升、规划管控提升、长效机制提升“六大行动”，建成一批“美丽桂林·幸福乡村”示范村，实现农村生活污水处理设施覆盖率、生活垃圾无害化处理率、无害化卫生厕所普及率明显提高。

　　实施公共服务能力提升专项行动。统筹推进乡村教育、文化、卫生、体育等公共服务设施项目，实现乡镇公办中心幼儿园全覆盖，村级公共服务中心覆盖率达85.9%。进一步健全乡村养老、社会福利服务体系。健全自治法治德治相结合的乡村治理机制，完善县、乡、村三级综治中心功能，发挥农村基层组织作用，提升乡村治理水平。培育文明乡风，留住乡韵、记住乡愁。

　　(四)提升旅游品质，增强三产活力

　　全力推动旅游高质量发展。确保年接待游客量、旅游总消费分别增长10%、15%。深入实施“旅游+”战略，大力发展红色旅游、健康旅游、文化旅游、研学旅游、体育旅游等新业态，加快推进全州天湖国际高山生态旅游度假区(一期)、全州大碧头国际旅游度假区、恭城瑶家大院互联网影视旅游基地等项目建设，打造桂林休闲旅游度假天堂。持续推进阳朔、秀峰等10个国家及自治区级全域旅游示范区创建，重点推动灵川、恭城创建广西特色旅游名县，加快打造覆盖大桂林的生态休闲旅游精品线路，实现南北旅游互动发展，促进全域旅游扩面升级。突出抓好品牌引进与培育，大力推动名城名企合作，推进旅游产品国际化、品牌化，着力引进四季酒店等一批国际品牌酒店、购物商店，加快推进阳朔洲际酒店等项目建设，支持阿丽拉、喜来登等酒店做大做强，培育壮大旅发展、旅股份、榕湖饭店等一批现有骨干企业，加速形成国际品牌聚集效应。突出抓好景区景点提升，大力整合桂阳公路沿线景区景点资源，打造旅游新“航空母舰”；持续提升猫儿山养生度假区等景区品质，推进遇龙河、桃花湾创建国家级旅游度假区；将东西巷、逍遥楼纳入王城5A级景区，丰富景区内涵。突出抓好旅游服务升级，持续推进龙脊梯田景区大循环公路等项目建设，加快建设城镇连接乡村、干线连通景区的“运动桂林·健康绿道”；建设磨盘山旅游集散中心，开通主要交通枢纽、旅游集散中心与景区之间的直通车，探索将高速公路服务区打造成旅游房车宿营地；加快旅游咨询服务中心、旅游驿站、旅游标识标牌和景区无障碍设施建设，建成旅游厕所100座以上；增加“i游桂林”用户数量，扩大平台覆盖面，打造一批智慧景区、智慧酒店，推动智慧旅游升级发展。突出抓好旅游市场环境综合监管，开展景区、旅行社、导游、餐饮店和商店信用等级评定，打造桂林旅游信用城市品牌；推广使用电子行程单，整合完善统一的旅游投诉平台，提升旅游投诉受理的权威性、时效性。突出抓好旅游营销推介，深化与国内外知名社交平台、媒体和线上线下旅行商合作，策划实施系列促销活动，拓展国内外市场；加强旅游商品开发，大力营销“桂林有礼”品牌，持续扩大旅游消费；继续办好“两会一节”[18]等重大活动，提升旅游品牌形象。

　　大力推进服务业提质增效。提升生产性服务业，加快大溪河物流园、桂林西货运枢纽物流园、珠子洲物流园区货运码头建设，建成现代物流大数据中心，推动流通服务业转型发展；鼓励金融机构创新产品与服务，支持桂林银行、国海证券等企业健康快速发展；开工建设新会展中心，大力培育会展龙头企业，引进更多国际会议在桂林定期召开，打造区域性会展中心和会议目的地城市；大力发展中介、广告、租赁、设计、咨询等商务服务业。发展生活性服务业，加快推进正阳东西巷、华润万象城、阳朔乌布小镇等商业综合体和叠彩百亿现代商贸园区建设，培育发展一批城市核心商圈；建立和完善租购并举住房制度，积极培育住房租赁市场；继续支持桂林航空、星达家电、桂林宾馆、南城百货、电信桂林公司、移动桂林公司、联通桂林公司、铁塔桂林公司等重点企业做大做强。加快服务业集聚区建设，推动桂林国家大学科技园、桂林袭汇国际文化世界等38个服务业集聚区加快发展，加快城区老厂房等存量资源再开发，促进形成新的服务业集聚区，新增2—3个自治区级服务业集聚区。新培育规上限上服务业企业100家以上，新认定自治区级服务业龙头企业10家以上。

　　加快打造百亿米粉产业。规范使用“桂林米粉”国家地理标志证明商标，完善桂林米粉全产业链标准体系。培育壮大三养易食等米粉预包装企业，启动桂林米粉产业园建设，打造集原料供应、生产研发、检验配送、旅游体验为一体的现代产业综合体。推进桂林米粉品牌店升级改造，认定一批米粉原材料基地，挖掘和重振老字号品牌，评选推广一批新品牌。加快建设桂林米粉中央厨房和配送中心，推动桂林米粉走出桂林、走出广西、走出国门。加强与知名电商合作，布局线上线下销售网络，开展“五进五出”[19]行动，加速桂林米粉无人售粉机投放与推广，扩大桂林米粉产业规模。

　　(五)聚焦数字桂林，培育发展新动能

　　加快数字桂林建设，培育一批新兴产业，形成一批新的投资需求，催生一批新的消费热点，打造经济发展新引擎，助推桂林发展“弯道超车”。

　　加快完善数字基础设施。持续推进“宽带桂林”建设，实施“千兆城区、百兆乡村”光网工程，大力开展老城区光纤到户改造，实现行政村4G信号覆盖率达100%。推进新一代移动互联网建设，重点推进4G网络优化，打造桂阳公路数字大道；推动桂林率先进行5G试用商用。推进通信基础平台建设，完成华为云计算数据中心主机房建设；加快整合行业部门数据资源，打造统一的政务云、企业云平台。

　　加快推动数字产业化。大力发展大数据、云计算、物联网、软件和信息服务业，加快推进华为信息生态产业合作区、高铁园智慧产业园孵化中心等项目建设，推动深圳华谊智测等项目投产运营，深化与华为、腾讯、东盟信息港等知名企业战略合作，积极引进关联企业落户。大力发展电子信息产品制造业，加快推进光隆光电激光器芯片项目(二期)、智神电子三轴手持稳定器产业化基地、飞宇智能摄像稳定器产业园等项目建设。大力推进动漫游戏与旅游、教育、文化等产业融合发展，支持力港、坤鹤等企业开拓海外市场。加快布局人工智能、区块链、北斗导航等新兴前沿数字产业。

　　加快推动产业数字化。打造桂林智能制造城，加快建设一批智能工厂、数字化车间、自动化生产线，实施福达曲轴智能工厂、三金现代中药产业智能化改造工程等一批试点示范项目。持续开展两化融合管理体系试点推广应用，推进规模企业上云，积极培育一批个性化定制示范企业。加快农业数字化发展，实施数字化现代特色农业示范区建设工程，大力推广水肥一体化、远程视频监控等技术。加快服务业数字化发展，推进国家电子商务示范基地、国家电子商务进农村综合示范县建设；加快建设统一的健康养生养老大数据平台，支持智慧康养、智慧物流等产业快速发展。

　　加快数字社会和数字政府建设。推动大数据、互联网技术在民生领域广泛运用，大力发展智慧城管、智慧交通、智慧医疗、数字社区。加快推进“互联网+政务服务”建设，实施政务数据“聚通用”攻坚行动，加快建成统一规范、双向互动、安全高效的政务服务体系。

　　(六)统筹城乡建设，扩大有效投资

　　坚持以项目建设为抓手，统筹推进城乡协调发展，实施市级统筹推进重大项目990项以上，年度计划投资增长10%以上。

　　加快交通基础设施建设。建成阳朔至鹿寨、贺州至巴马(平乐段)高速公路，推进荔浦至玉林、桂林至柳城、灌阳经恭城至平乐等高速公路项目，建设改造一批国省干线公路；推进怀桂铁路(桂林段)、龙胜至湖南(城步)高速公路、巴江口船闸改扩能工程前期工作；完成桂林西站改扩建，推进桂林站、桂林北高铁枢纽改造提升。

　　完善新区城市功能。抓好临桂新区规划提升工作。加快机场路以北片区开发，推动连接新区老城的轨道交通建设。加快宝湖书院、崇文小学、宏谋中学、新城中学等一批学校建设。建成启用桂林旅游综合医院，提升扩建桂林医学院第二附属医院。推进临桂万达广场、吾悦广场、宏谋市场、枫林市场建设。加快兴桂园、新城商务写字楼项目建设，实现市公安局技术大楼、广电中心、桂林日报社传媒中心入驻使用。加快建设义江引水、大皇山污水处理厂工程(二期)，推进北区水系建设。做好核心区水系、标志性建筑、城市主干道美化亮化，提升新区宜居水平和形象品位。

　　提升老城城市品质。持续推进特色街区改造，完成靖江王府片区历史文化旅游休闲街区改造提升工程，推进漓江城市段两岸片区改造提升，加快福隆园、新生街、塔山片区棚户区等项目建设。推进琴潭千亩荷塘湿地项目。加快实施“畅通缓堵”工程，加强重要节点改造，有序打通一批断头路，进一步改善老城区交通条件；完成八一桥改扩建、东西巷与正阳步行街地下通道等项目；加快推进综合运输服务示范城市、公交都市创建工作，推进停车资源共享利用，缓解停车难问题。提升城市业态，妥善利用老城区腾出空间，重点发展文化旅游、楼宇经济、“互联网+”等特色产业，促进老城区业态调整与产业升级，打造功能完善、特色鲜明、文化品位突出的城市景区。

　　加强和创新城市治理。开展国土空间规划研究，严格城市规划管理，加强控制性详细规划的公开性和强制性，严格控制建筑高度、密度、容积率；统筹好城市水域、绿地、文化、业态、休闲五大要素空间布局，按照“显山、露水、透绿、美城”的要求，加快打造公园城市。加强城市设计，注重城市风貌整体性、文脉延续性，精雕细琢一砖一瓦，做到建成一项工程、留下一个亮点、增加一个精品。扎实做好控违拆违工作，坚持一级抓一级、层层抓落实，健全控违长效机制和违建惩戒机制，各城区要组建专门巡查队伍，实行全天候、全覆盖、不间断巡查，坚决遏制违法建设。提升城市管理精细化水平，积极推广装配式建筑，力争全年建筑装配化率达15%；坚持最严格的城市管理考评，切实发挥基层组织作用，坚持严管重罚，严格落实“门前三包”，加强道路绿化管护，打造干净有序的美丽街区，争创与山水同美的国家文明城市。

　　全力推动县域经济高质量发展。按照2018年县域经济发展乡村振兴推进大会的部署要求，推动各县(市、区)按照主体功能区划分，争创广西科学发展先进县、进步县。深入实施大县城战略，加快荔浦城市转型，做大做强现代工业；加快灵川与中心城区一体化发展，打造桂林北新城；按城市标准，加快全州、兴安、平乐城镇化步伐，做大产业规模，加快推进全州撤县设市；加快阳朔新区建设，发挥全域旅游龙头作用；巩固龙胜、资源、灌阳脱贫攻坚成果，强化资源整合，促进生态旅游与特色产业融合发展；加快恭城产业转型升级，推进生态农业与康养产业、文化旅游融合发展；临桂、永福要服务新区、经开区，加快产城互动发展。

　　深入推进示范乡镇建设。加快补齐新型城镇化示范乡镇民生短板，整体推进集镇产业、基础设施、公共服务、生态环境优化升级，完成第四批、启动第五批示范乡镇建设。

　　(七)生态文化相融，建设大美桂林

　　坚持生态立市、文化强市，持续打响桂林历史文化名城和生态山水名城品牌，努力将生态文化优势转化为发展优势。

　　推进漓江生态保护和修复。深化漓江“三统”改革，完善漓江管理体制，推动出台《桂林漓江风景名胜区条例》；推动漓江生态保护和修复提升工程、漓江流域山水林田湖草(江)项目列入国家项目库。加强漓江上游水源林保护，增强水源涵养功能。开展漓江环境综合整治，加快推进漓江截污工程(三期)，持续实施“四乱一脏”专项整治，巩固风景名胜区内关停采石场复绿成果。开工建设第二水源等项目，完成长塘水库前期工作，加快实施漓江城市段洲岛修复工程，完成沿江码头改造提升和慢行步道建设。发挥漓投公司作用，推进漓江保护重大项目建设。

　　打好污染防治攻坚战。严格落实党政同责、一岗双责，全力抓好中央环保督察反馈意见整改，坚决打赢蓝天、碧水、净土保卫战。大力推进大气污染防治，实施工业企业污染综合治理工程，强化面源污染和扬尘综合治理，确保市区空气质量优良天数完成自治区下达目标任务。大力推进水污染防治，扎实推进河(湖)长制，加强饮用水水源地保护区规范化建设，加快建设县(市、区)工业集聚区污水集中处理设施，确保主要河流考核断面水质达标率100%。大力推进土壤污染防治，开展土壤污染详查，加快重金属及土壤污染防治项目建设，严厉打击固体废物非法转移等违法犯罪行为。

　　加强生态系统建设。加强石漠化治理，切实保护生态公益林、水源林。强化外来入侵物种综合防控。加快提升猫儿山、花坪、千家洞、银竹老山等国家自然保护区功能，完成自治区下达的植树造林任务，全面提升国土绿化水平。推进国家低碳城市试点建设，倡导绿色低碳生活方式，积极推进节能环保、清洁生产、清洁能源发展，大力发展绿色循环经济。

　　加强文化建设。做好湘江战役红军遗骸收殓保护，完成酒海井、光华铺等红军遗骸集中安放点改扩建；抓好红色设施修缮保护，建成红军长征湘江战役纪念公园，做好新圩阻击战陈列馆、湘江战役纪念馆等纪念设施展陈提升，推进国家长征文化公园和国家重点红色旅游景区建设；深度挖掘红色故事、红色记忆，创作一批主题文艺作品，推出一批主题展览，传承发扬红色精神。坚持“寻找桂林文化的力量，挖掘桂林文化的价值”，将文化资源与旅游、生态有机融合，推出一批文学影视作品，打造一批景区景点；加快桂林文化旅游中心建设，推进桂海碑林博物馆改造和东巷博物馆布展开馆，修缮省立艺术馆，建设非遗展示馆，推进灵渠申报世界文化遗产。加快发展公共文化事业，深入实施文化惠民工程，持续开展“百姓大讲坛”等群众性品牌文化活动，推动《桂林有戏》等文化精品“走出去”。深入开展“扫黄打非”。加快文化产业创新发展，推进象山文化产业园等项目建设，支持广西师大出版社等企业发展壮大。

　　(八)深化改革开放，营造一流环境

　　深化“放管服”改革。积极推行以企业投资项目建设审批“容缺后补、容错纠错，企业向政府承诺、政府向企业承诺”为主要内容的“双容双承诺”改革，变投资项目“先批后建”为“先建后验”，实现投资项目“直接落地”。大力实施政务服务“一网通办“一事通办”改革，建成全市一体化网上政务服务平台并接入市政务云，实现政务服务“一张网、一朵云”[20]，加快推进“不见面审批”，实现审批全过程留痕、全流程监管，杜绝权力寻租，确保高效透明。继续深化商事制度改革，全面推行“证照分离”“多证合一”，大幅减少行政审批，着力解决“准入不准营”问题。全面落实自治区“354560”[21]提速行动，实现企业开办、不动产登记、投资项目审批再提速。

　　深化重点领域改革。按照“巩固、增强、提升、畅通”方针，深入推进供给侧结构性改革，加快淘汰落后产能，培育壮大高质量有效供给，有序化解非住宅商品房库存，切实减轻企业负担，稳妥降低杠杆率。规范有序推进市县机构改革，着力构建精简高效的行政管理体制。积极推动“多规合一”[22]、旅游资源整合。继续深化城市管理、国资国企、财政管理体制、城市公立医院等领域改革。深入开展农村集体资产清产核资与股权管理改革，持续推动农村土地承包经营权确权登记颁证工作，深化林权制度改革。全力做好第四次全国经济普查，持续推进新时代基层统计工作规范化建设。

　　大力支持民营企业发展。实施强优民营企业扶持计划，落实支持民营企业发展系列政策，让民营企业真正成为稳投资的主体。用好城镇低效用地再开发政策，结合老城区闲置资产处置，谋划推出一批产业项目，重点引导民营企业参与开发，激发民营企业投资活力。给予民营企业家更多的关心爱护，着力营造尊商重商亲商的社会环境。

　　防范化解重大风险。千方百计培植税源，加强土地收储出让，盘活存量资产，增加财政收入。落实全面预算绩效管理，压缩一般性支出，做到“花钱必问效、无效必问责”。严控政府债务风险，积极合法合规增信，有效化解存量债务，确保全市债务处在合理水平。推动国有投融资公司转型发展，加快推进投融资公司存量债务置换，尽快去平台化、去行政化，实现市场化转型；选择一批条件较好的公司，整合优质资源、注入优质资产，形成稳健主营业务及良好现金流，提升经营创收能力，为全市重大基础设施建设多作贡献。强化金融监管，严控不良贷款风险，坚决打击非法集资、恶意逃废债务、金融诈骗等行为，牢牢守住不发生系统性区域性金融风险的底线。

　　实施创新驱动发展。加快科技创新平台建设，引导企业与高校、科研院所对接，推动政产学研合作，力争新增自治区级以上创新平台和基地5家以上。统筹各类专项资金，大力支持开展核心技术攻关，推动科技创新成果就地转化。围绕产业转型升级、新动能培育的关键领域，大力引进一批高层次人才。

　　提高对外开放水平。主动融入“一带一路”、中国—东盟自贸区、泛珠三角、珠江—西江经济带建设，依托中国—东盟博览会“两会一节”等平台开展经贸合作。办好第五届粤桂黔高铁经济带合作联席会议。充分发挥地缘相近、高铁联结优势，把粤港澳大湾区作为开放合作的重点，加密与大湾区及周边省会城市的航线，主动承接产业转移，力争全年招商引资内外资合计到位资金800亿元。

　　(九)坚持精准脱贫，实现共享发展

　　坚决打赢精准脱贫攻坚战。把脱贫攻坚作为最大的政治责任、最大的民生工程和最大的发展机遇，聚焦“两不愁、三保障”[23]，按照“八有一超”[24]“九有一低于”[25]“十一有一低于”[26]标准，全力打好“五场硬仗”，持续开展“六大行动”，确保灌阳脱贫摘帽，104个贫困村、4.3万贫困人口脱贫出列，龙胜、资源脱贫摘帽通过国家复核。着力破解深度贫困问题，逐乡逐村制定攻坚方案，进一步加大政策扶持力度，集中力量决胜全面脱贫奔小康。持续推进产业扶贫，力争实现贫困户全覆盖，确保每个贫困村村级集体经济收入超4万元。持续推进贫困地区基础设施建设，重点补齐贫困村村屯道路、宽带网络等短板。持续推进易地扶贫搬迁安置，大力支持建设扶贫车间，实现搬迁户就近就业、稳定发展。持续推进健康、教育扶贫，加大贫困学生资助力度，实现建档立卡户子女义务教育阶段零辍学，做到“培养一名学生，带富一个家庭”。深入推进肇庆—桂林扶贫协作，广泛动员社会力量参与扶贫。坚持问题导向，认真做好扶贫领域上级巡视督查检查反馈和自查自纠问题整改，不断提升脱贫攻坚工作质量与水平。

　　全面发展民生社会事业。大力实施教育提升三年行动计划，年度投资10.5亿元，新建中小学校(幼儿园)30所，改扩建228所，实现国家县域义务教育发展基本均衡县(市、区)全覆盖。深化义务教育学区制管理改革，破解地域性学位紧张和“择校热”问题。推进产校融合，鼓励榕湖小学、桂林中学等优质学校到园区开展集团化办学。强化校外培训机构整治，促进民办教育规范健康发展。推进中等职业学校布局调整、专业结构优化。持续推进高校集聚区建设，加快建设花江智慧谷电子信息创业产业园，建成芳香东路；加快雁山大学集中区大学生创业产业园、“南北飞翔”4条道路建设，建成桂林理工大学雁山校区东大门人行天桥，推动雁山人民医院尽快投入使用。支持桂林师专“专升本”。大力实施健康桂林建设，加快医联体建设步伐，提高医疗服务质量，提升公共卫生服务水平，加快中医药事业健康发展，促进生育政策和经济社会政策配套衔接。大力开展全民健身运动，办好环广西公路自行车赛、桂林国际马拉松赛等重大活动，力争漂流世锦赛尽快落户资源。

　　抓好就业和社会保障。实施就业优先政策，推进创业孵化基地建设，确保完成自治区下达的城镇新增就业、城镇失业人员再就业、农村劳动力转移就业任务。实施全民参保计划，完善城乡社会救助体系，稳步提高最低生活保障水平。

　　打造共建共治共享社会治理格局。深入实施“雪亮工程”，扎实开展星级平安小区(村屯)创建活动，打造“平安桂林”升级版。深入开展扫黑除恶治乱，加大打击传销力度，严厉打击征地拆迁、违法建设、交通运输、工程建设等领域涉黑涉恶行为，持续强化禁毒严打整治，广泛动员群众参与社会治安综合治理。抓好防灾减灾、安全生产、市场监管等工作，积极创建广西食品安全示范城市。

　　深入开展国防教育，积极做好国防动员、人民防空、民兵预备役和双拥、优抚安置等工作，争创自治区双拥模范城十连冠；加强退役军人服务管理，推进军民融合深度发展。

　　继续实施为民办实事工程。坚持财政支出更多向民生倾斜，确保完成自治区和我市为民办实事工程任务，让发展成果更多惠及百姓。

　　各位代表！为人民服务、对人民负责、受人民监督、让人民满意，是政府的职责与使命。我们一定坚持以全面从严治党统领政府各项工作，更加奋发有为、勤勉务实，切实提升管理服务效能，使政府工作赢得人民群众更多的信任和支持。

　　——强化责任担当。牢固树立“实干就是能力，落实才是水平”的理念，不断增强政治担当、历史担当、责任担当和行动自觉。

　　——强化法治理念。坚持依法行政，进一步规范重大行政决策的范围、认定标准、决策程序等内容。自觉接受市人大及其常委会法律监督、工作监督和市政协民主监督，主动接受社会监督和舆论监督，认真听取各民主党派、工商联、无党派人士和人民团体的意见，全力办好市人大代表建议、市政协提案。

　　——强化服务意识。坚持为人民服务的宗旨，深化行政体制改革和职能转变，努力提高为民办事效率，建设人民满意的服务型政府。

　　——强化精品意识。牢固树立质量就是生命的理念，杜绝浮躁之风，坚持久久为功，做到每一个决策、每一件工作、每一项工程都务求实效，力求最好，经得起历史的检验，经得起群众的评判。

　　——强化廉洁建设。把加强政治建设作为政府系统党的建设的首要任务，严格执行中央八项规定及其实施细则精神，深化集中整治形式主义、官僚主义成果，严查重点领域特别是涉及群众切身利益的腐败问题，深化标本兼治，为全市经济社会持续健康发展提供坚强纪律保证。

　　各位代表！征程万里风正劲，重任千钧再扬鞭。让我们高举习近平新时代中国特色社会主义思想伟大旗帜，牢固树立“四个意识”，坚定“四个自信”，践行“两个维护”，更加紧密地团结在以习近平同志为核心的党中央周围，在自治区党委、政府和市委的正确领导下，不忘初心、牢记使命，为实现桂林“两个建成”目标，建设壮美广西、共圆复兴梦想作出新的更大贡献，以优异成绩庆祝新中国成立70周年！
